# Hepatocellular carcinoma in the absence of liver cirrhosis in a treated hepatitis C virus patient

**DOI:** 10.4103/0256-4947.51789

**Published:** 2009

**Authors:** Omar M. Al Nozha, Hamad Al Ashgar, Mohammed Khan, Hadeel Al Mana

**Affiliations:** a From the Department of Medicine, King Faisal Specialist Hospital and Research Centre, Riyadh, Saudi Arabia; b From the Department of Pathology and Laboratory Medicine, King Faisal Specialist Hospital and Research Centre, Riyadh, Saudi Arabia

**Figure 1:**
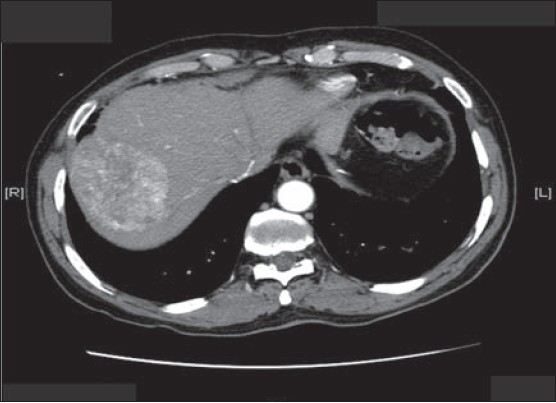
Triphasic CT scan of the abdomen showing a liver mass at segment 5 and 8; a) arterial phase, b) portal-venous phase.

**Figure 2:**
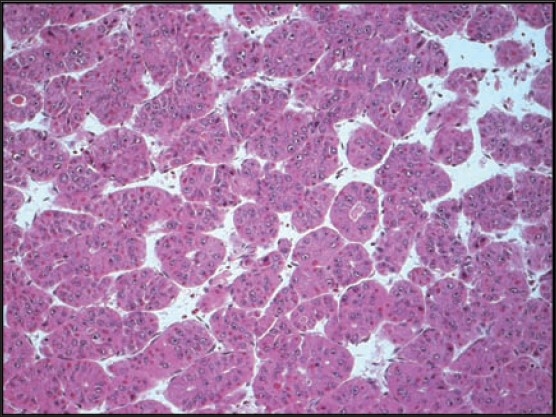
Hematoxylin and eosin stain of the HCC tissue showing classical pseudo-glandular and trabecular pattern.

**Figure 3:**
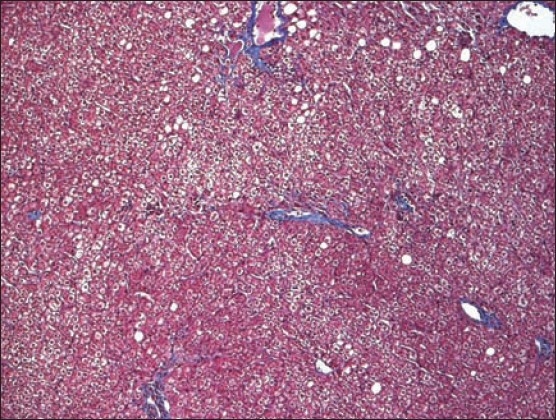
Trichrome stain of the non-neoplastic liver tissue only mild steatosis and no evidence of cirrhosis.

**To the Editor:** A 65-year-old male patient was referred to King Faisal Hospital and Research Centre after he presented with jaundice, fever, abdominal discomfort and biochemical evidence of chronic hepatitis C virus (HCV) hepatitis with no clinical or histological evidence of cirrhosis. HCV polymerase chain reaction (PCR) was positive and HCV quantitation was 4682 copies/mL. Liver function test (LFT) results were ALT 225 IU/L (reference range, 5-60 IU/L), AST 104 IU/L (reference range, 5-43 IU/L), total bilirubin 20 μml/L (reference range, 5.1-17.0 μmol/L), INR 1.2 (reference range, 0.9-1.1), and albumin 38 g/L (reference range, 35-50 g/L). Liver biopsy showed chronic hepatitis grade II, stage II portal fibrosis and no evidence of cirrhosis. Alpha-fetoprotein was 6 μgm/L. The patient completed a full year of combination therapy of pegulated interferon 3 million units subcutaneously 3 times/week with ribavirin 1000 mg orally daily and achieved a negative viral PCR with normal LFTs. He had 3 years of follow-up with a sustained virological response (sustained loss of detectable virus in response to antiviral therapy) and was then discharged from our clinic and instructed to follow-up at his local hospital. A CT scan at discharge showed no evidence of cancer (image not available). Two years later he was referred back from his local hospital with a huge right hypochondrial mass associated with pain but no jaundice, fever or weight loss. The mass was about 10 cm in diameter, difficult to separate from the liver, ill-defined, lobulated and hard. Basic tests, including CBC and renal profile were normal. ALT 27 IU/L (reference range, 5-60 IU/L), AST 35 IU/L (reference range, 5-43 IU/L), ALP 75 IU/L (reference range, 30-120 IU/L), total bilirubin 14 μml/L (reference range, 5.1-17.0 μmol/L), INR 1.0 (reference range, 0.9-1.1), and albumin 42 g/L (reference range, 35-50 g/L). AFP was 2.3 μgm/L and carcinoembryonic antigen (CEA) was 2.1 μgm/L. Ultrasound of the abdomen showed a huge liver mass (8cm×11cm) and the right portal vein branch was not visualized distally. CT of the abdomen showed the same mass at segment 5 and 8 which was consistent with hepatocellullar carcinoma (HCC) showing the classical triphasic pattern, i.e., heterogenous enhancement in the arterial phase, but early rapid washout in the portal-venous phase leaving the HCC lesion hypo-dense compared to the surrounding normal tissue (Figure 1). The patient underwent surgical removal of both segments (5 and 8). Histopathology of the mass confirmed the diagnosis of HCC. H&E stain showed the HCC pseudoglandular and trabecular pattern (Figure 2) and extensive lymphovasculer invasion. Immunohistochemical stain for CD34 was positive and showed wrapping of the tumor cluster by endothelial cells. The rest of the non-neoplastic livers showed only mild steatosis and no cirrhosis at all (Trichrome stain) (Figure 3).

HCC, which arises from the hepatocytes, is the most common type of primary liver malignancy. It commonly arises in cirrhotic liver. Chronic HCV is a leading cause of cirrhosis worldwide.^1^ AFP levels may be normal in more than one third of patients.^2^ In our patient, despite having no evidence of cirrhosis and achieving a sustained viral response for 5 years after treatment with combination therapy, he developed HCC. In addition, he had a negative AFP screening test on more than one occasion. Okanoue et al^3^ conducted a long-term follow-up study of 1246 cases of chronic HCV who received standard pegulated interferon therapy to study the effect of interferon on development of HCC. Interferon lowered the rate of HCC development in both sustained and transient biochemical responders compared to none responders. but did not prevent HCC. In another study by Omata et al^4^ that examined the role of interferon treatment on prevention of HCC in chronic HCV patients, development of HCC was strongly associated with stage of liver fibrosis, age and gender. The risk was reduced to 1/5 by interferon, but not prevented among sustained responders compared to untreated patients. Patients at risk of developing HCC should be offered surveillance ultrasound at 3-4 monthly intervals (based on tumor doubling time).^5^ A negative initial screening for HCC does not mean that HCC is absent, but only that the tests are insufficiently sensitive. An abnormal screening test requires a triphasic CT or MRI or both, and failure to confirm the diagnosis requires enhanced CT at an interval of 3-4 months.^5^

We found only one case in the literture of HCC in the absence of cirrhosis, but the patient had an additional risk factor, which was porphyria cutanea tarda.^6^ An epidemiological study reported HCC with HCV in the absence of cirrhosis in 7 patients, but 5 had coinfection with HBV and the rest had evidence of high alcohol intake (alcohol intake >80 g/day).^7^ To our knowldge, our case is the first in a patient with no co-infection and no evidence of other risk factors for HCC apart from HCV infection. Our case suggests that HCC can develop years later in patients with HCV who were treated with interferon-alpha and achieved sustained virological response. It also indicates that normal AFP does not exclude HCC and therefore imaging studies should be used for surveillance for HCC.
